# Advocacy and Palliative Care as a Framework for Dignified Dementia Care in African Settings

**DOI:** 10.1002/alz70860_102718

**Published:** 2025-12-23

**Authors:** Zipporah Vunoro Ali

**Affiliations:** ^1^ Kenya Hospices and Palliative Care association (KEHPCA), Nairobi, Kenya; Alzheimer Dementia Organization‐Kenya, Nairobi, Kenya

## Abstract

Palliative care offers specialized care /support to persons with advanced disease, including Older Persons living with dementia related problems. The focus of care is on comfort, dignity and improving the quality of life through an approach that aims to prevent and relieve distressful symptoms and ill being through early planning, identifying and providing practical, emotional, psychological and spiritual care to both the patient and their family, carers and other loved ones. Additionally, palliative care guarantees the older person's dignity and respect for their final choices, aids in advanced care planning, and should be included into dementia care by beginning early to ensure the older person's participation in decision‐making. Currently, most of the palliative care in Africa focuses on patients with oncological problems. Unfortunately, there are no frameworks, policies or guidelines on providing palliative care for older persons with Dementia and neurodegenerative conditions. The main barriers to accessing palliative care for older patients with neurodegenerative conditions include lack of awareness among policy‐makers, health professionals on the benefits of palliative care. Other barriers are cultural and social around death and dying & misconceptions including that palliative care is only for dying patients, or only for cancer patients. To improve the quality of life for older patients (their families/carers) affected with neurodegenerative conditions there is an urgent need for advocacy at all levels of care to incorporate palliative care, including end‐of‐life care. Even though the person we knew and loved is no longer with us, the person we currently know and love is still important and deserving of a respectable existence. Therefore, we must advocate for quality palliative and End of Life services, policies and guidelines/frameworks that ensure our vulnerable elder populations are not left behind. Patients and family members/caregivers who have lived the experience should be included in advocacy efforts. It is also essential to engage the community. This session will discuss how we may collaborate with stakeholders to ensure access to high‐quality palliative care for older persons with neurodegenerative conditions.

